# Nusinersen demonstrates effectiveness in treating spinal muscular atrophy: findings from a three-year nationwide study in Korea

**DOI:** 10.3389/fneur.2023.1294028

**Published:** 2023-12-20

**Authors:** Jaeso Cho, Jiwon Lee, Jihye Kim, Hyunjoo Lee, Min-Jee Kim, Yun Jeong Lee, Mi-Sun Yum, Ji-Hye Byun, Chong Guk Lee, Young-Mock Lee, Jeehun Lee, Jong-Hee Chae

**Affiliations:** ^1^Department of Genomic Medicine, Seoul National University Children’s Hospital, Seoul, Republic of Korea; ^2^Department of Pediatrics, Samsung Medical Center, Sungkyunkwan University School of Medicine, Seoul, Republic of Korea; ^3^Health Insurance Review & Assessment Service (HIRA), HIRA Research Institute, Wonju, Republic of Korea; ^4^Department of Pediatrics, Gangnam Severance Hospital, Yonsei University College of Medicine, Seoul, Republic of Korea; ^5^Department of Pediatrics, University of Ulsan College of Medicine, Asan Medical Center, Seoul, Republic of Korea; ^6^Department of Pediatrics, Kyungpook National University Hospital, Kyungpook, Republic of Korea; ^7^Department of Pediatrics, Seoul National University College of Medicine, Seoul, Republic of Korea

**Keywords:** spinal muscular atrophy, nusinersen, Spinraza, long-term effect, early treatment

## Abstract

**Introduction:**

Nusinersen is the first drug approved for spinal muscular atrophy (SMA) treatment. In this study, we aimed to evaluate the long-term safety and efficacy of nusinersen, assess the therapeutic effects based on the treatment initiation timing and baseline motor function, and explore the perception of functional improvement from either parents or patients, utilizing 3-year nationwide follow-up data in South Korea.

**Methods:**

We enrolled patients with SMA who were treated with nusinersen under the National Health Insurance coverage, with complete motor score records available and a minimum treatment duration of 6 months. To evaluate the motor function of patients, the Hammersmith Infant Neurological Examination-2 (HINE-2) was used for type 1 and the Expanded Hammersmith Functional Motor Scale (HFMSE) was used for types 2 and 3 patients. A significant improvement was defined as a HINE-2 score gain ≥5 for patients with type 1 and an HFMSE score **≥** 3 for patients with types 2 and 3 SMA. Effects of treatment timing were assessed. Patients with type 2 were further categorized based on baseline motor scores for outcome analysis. We also analyzed a second dataset from five tertiary hospitals with the information on parents/patients-reported impressions of improvement.

**Results:**

The study comprised 137 patients, with 21, 103, and 13 patients representing type 1, 2, and 3 SMA, respectively. At the 3-year follow-up, the analysis encompassed 7 patients with type 1, 12 patients with type 2, and none with type 3. Nearly half of all enrolled patients across SMA types (42.8, 59.2 and 46.2%, respectively) reached the 2-year follow-up for analysis. Patients with type 1 SMA exhibited gradual motor function improvement over 1-, 2-, and 3-year follow-ups (16, 9, and 7 patients, respectively). Patients with type 2 SMA demonstrated improvement over 1-, 2-, and 3-year follow-ups (96, 61 and 12 patients, respectively). Early treatment from symptom onset resulted in better outcomes for patients with type 1 and 2 SMA. In the second dataset, 90.7% of 108 patients reported subjective improvement at the 1-year follow-up.

**Conclusion:**

Nusinersen treatment for types 1–3 SMA is safe and effective in long-term follow-up. Early treatment initiation was a significant factor affecting long-term motor outcome.

## 1 Introduction

Spinal muscular atrophy (SMA) is an autosomal recessive neuromuscular disease characterized by progressive muscular atrophy and weakness resulting from apoptosis of the anterior horn cells of the spinal cord (1, 2). Its estimated incidence is 1 in 11,000 live births (1, 3). It is caused by mutations in the survival motor neuron 1 (*SMN1*) gene located on chromosome 5, which encodes the SMN protein (4). The SMN protein is essential for the survival of the anterior horn cells of the spinal cord. *SMN2*, a highly homologous *SMN1* gene located on chromosome 5, produces few functional SMN1 proteins through alternative splicing in exon 7. Patients with higher number of the *SMN2* gene copies experience milder and later-onset motor weakness owing to the relatively higher production of functional SMN1 protein through alternative splicing of *SMN2* (3, 5).

Nusinersen (Spinraza®) increases SMN protein production by modifying *SMN2* splicing. As the first drug approved for SMA treatment, clinical trials have demonstrated its potential in improving survival and motor function in infants and children with SMA types 1 and 2 (1, 6, 7). Subsequent to regulatory approval, many observational studies have reported its beneficial effects on motor function across all types of SMA (3, 7–10). Nevertheless, the limited number of population-based studies has prevented clinicians from referring to real-world data (11, 12).

South Korea has a unique cohort in which the entire cost of nusinersen is covered by the National Health Insurance, thus allowing us to enroll all patients with SMA in South Korea. Such data can provide real-world evidence of the effects of nusinersen and a nationwide population-based picture of nusinersen therapy. In this study, we present a 3-year follow-up of a population-based patient group with SMA types 1–3, who were treated at various stages of disease progression. Our objectives were to: 1) evaluate the long-term safety and efficacy of nusinersen across all SMA types, 2) assess the therapeutic effects of treatment initiation timing on motor function outcomes, and 3) provide real-world long-term data on the efficacy of nusinersen in different baseline motor function subgroups. Additionally, we sought to explore the perception of functional improvement from either parents or patients.

## 2 Patients and methods

### 2.1 Patients

Two datasets were used in the study. The first database was derived from the Korean Health Insurance Review and Assessment Service (HIRA) data, encompassing patient information for reimbursement between April 2018 and December 2021. The second dataset included data on perceptions of functional improvements reported by patients or parents from five tertiary hospitals in Korea. The inclusion criteria were as follows: a genetic diagnosis of 5q SMA, approval for nusinersen therapy by the HIRA, availability of complete data on motor function tests, (including both baseline and follow-up data), and a follow-up period of at least 6 months. Data on the date of birth, sex, age at diagnosis, SMA type, *SMN2* gene copy number, age at treatment initiation, ventilator support status, and motor function were retrospectively reviewed using HIRA records. SMA types 1–3 were defined based on the age at onset and acquired motor skills (13). This study was approved by the Institutional Review Board (IRB) of Seoul National University Hospital (IRB no. H-2011-179-1177), Korean HIRA (IRB no. 2022–047-002), and Samsung Seoul Hospital (IRB no. 2022–03–100-002). Informed consent was obtained from all the participants in accordance with the Declaration of Helsinki.

### 2.2 Reimbursement criteria of the national health insurance in Korea

The Korean National Health Insurance reimburses nusinersen therapy for patients with SMA based on the following criteria: a genetically confirmed diagnosis of SMA with either a deletion or mutation in the *SMN1* gene, symptom onset before 3 years of age, and no requirement for permanent respiratory support (<16 h/day and no consecutive 21 days of support). Owing to the reimbursement restrictions, no patients with SMA type 3B were included in our study. The timing of review for reimbursement is before the loading doses, at the 5th dose, and thereafter every subsequent injection at 4-month intervals. The review is done by Health Care Review and Assessment Committee of HIRA for National Health Insurance of South Korea. Motor and respiratory functions as well as adverse events were reviewed for insurance coverage. Reimbursement is halted if there is a need for permanent ventilation or if there is no improvement or maintenance of motor function test scores in two successive dose intervals without a definitive cause. Owing to lack of newborn screening in South Korea, no pre-symptomatic patients have received nusinersen treatment to date; consequently, none were included in the analysis.

### 2.3 Motor function assessment

To evaluate the motor function of patients with type 1 SMA, the HIRA requires the use of the Hammersmith Infant Neurological Examination-2 (HINE-2) (14). For patients with type 2 or 3 SMA, the Expanded Hammersmith Functional Motor Scale (HFMSE) is used (15). In cases where patients with type 2 or 3 SMA were unable to sit at the time of the initial nusinersen treatment, the HINE-2 was also used with the HFMSE. The HINE-2 and HFMSE scores were collected at baseline, month 2, and every 4 months thereafter (0, 2, 6, 10, 14, 18, 22, 26, 30, and 34 months) for submission for reimbursement approval. Annual follow-up assessments were conducted at 14, 26, and 34 months. In this study, we referred to these time points for the 1-, 2- and 3-year follow-up. A significant improvement was defined as HINE-2 score gain greater than or equal to 5 in patients with type 1 SMA and HFMSE score gain greater than or equal to 3 in patients with types 2 and 3 SMA.

To assess the motor function improvements based on the baseline motor state, patients with type 2 SMA were categorized into different subgroups based on their absolute HFMSE scores for further analysis. Patients with a baseline HFMSE score of 0 were assigned to the 0% group, 1–6 to the <10% group, 7–16 to the 10–25% group, 17–33 to the 25–50% group, 34–50 to the 50–75% group, and 51–66 to the >75% group. The patients were further grouped into “high” (baseline HFMSE score ≥ 35) and “low” (baseline HFMSE score < 35) categories to compare the long-term motor function outcome.

HINE-2 scores range from 0 to 26 and HFMSE scores ranges from 0 to 66, with higher scores indicating better motor functions.

### 2.4 Patient/parent-reported impressions of improvement and deterioration

The second dataset, derived from five tertiary hospitals in South Korea (Seoul National University Children’s Hospital, Gangnam Severance Hospital, Samsung Medical Center, Asan Medical Center, and Kyungpook National University Hospital), comprises the information on 115 patients (73.7%) from the first dataset. No new patients were enrolled in this analysis, and matching with the initial dataset was not performed owing to the anonymization of patient data, except for SMA types and baseline motor scores. This dataset incorporated information on patient/parent-reported impressions of improvement and deterioration in general function, respiration, fine motor function, and swallowing/speech. The data collected were based on three questions as follows: “did you observe any change in general function (stable, improved, or deteriorated)?”; “which function improved as reported by patients/parents (respiratory, fine motor function, swallowing, and speech)?”; and “did you observe any deterioration as reported by the patients/parents (respiratory, fine motor function, swallowing, and speech)?.” The final clinical follow-up was the assessment period.

### 2.5 Statistics

Descriptive statistics were used for data expressed as means (standard deviations) and percentages. Data analysis was performed using GraphPad Prism 9.3.1. The Mann–Whitney U test was used for non-parametric comparisons between the two groups. A simple linear regression analysis was performed to estimate the relationship between the independent and dependent variables. The *R^2^* value was used to evaluate the goodness of fit. Statistical significance was set at *p* < 0.05.

## 3 Results

### 3.1 Demographic and baseline clinical characteristics

**Figure 1:**
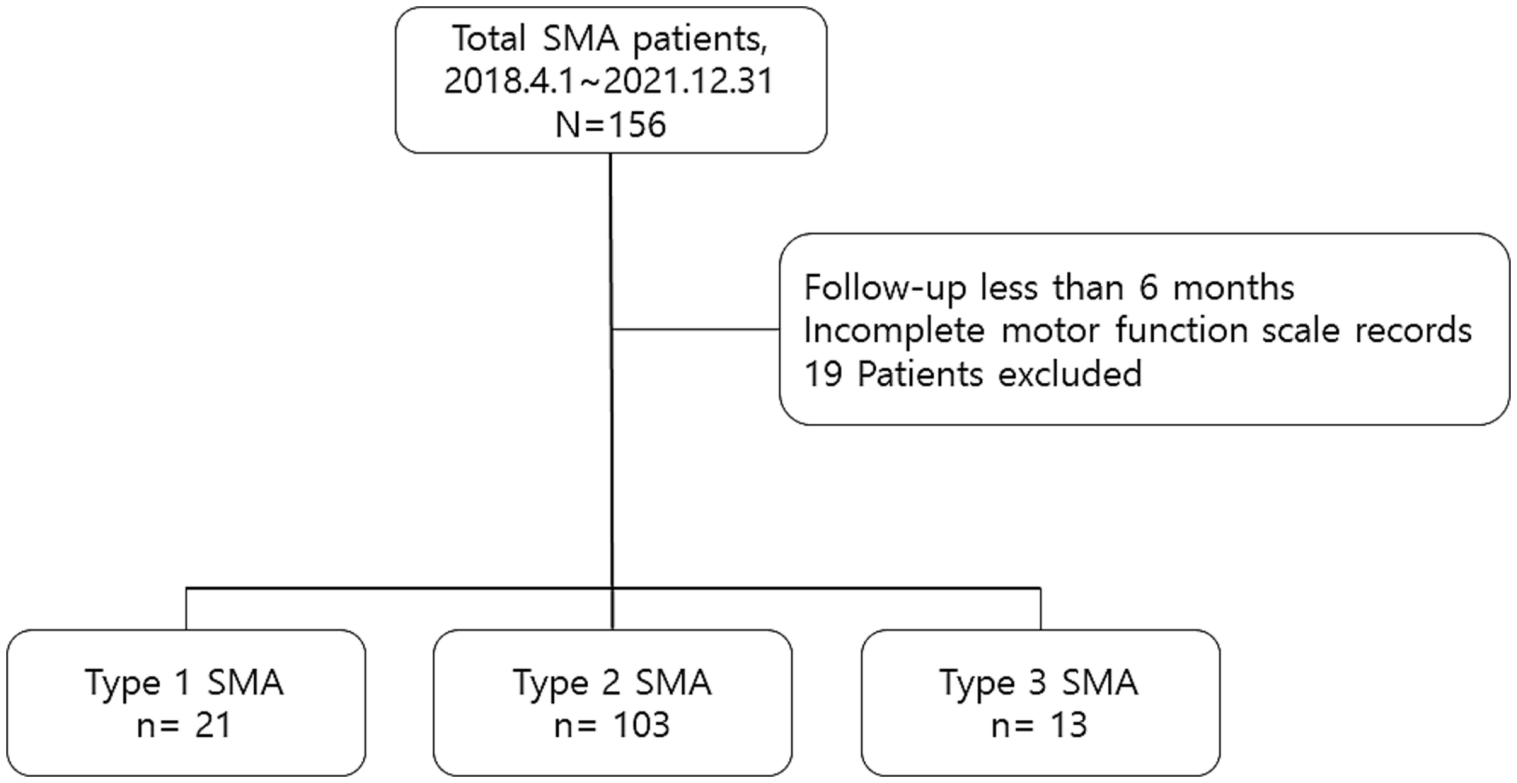
Overview of the study inclusion criteria.

The first dataset from the HIRA initially comprised 156 patients—25 with type 1 SMA, 116 with type 2 SMA, and 15 with type 3 SMA. After excluding patients who had been treated for <6 months (4 with type 1 SMA, 2 with type 2 SMA, and 1 with type 3 SMA) or those with missing functional measurement data (11 with type 2 SMA and 1 with type 3 SMA), the final dataset included 137 patients, comprising 21 with type 1 SMA, 103 with type 2 SMA, and 13 with type 3 SMA (Figure 1). All patients experienced symptom onset before the age of 3 years. At the time of injection, 46 patients were 18 years or older, constituting 33.6% of the cohort, with a mean age of 28.3 years (SD 7.2, range 18.5–47.7). Among adult patients, 1 had type 1 SMA, 36 had type 2 SMA, and 9 had type 3 SMA. No serious adverse events requiring treatment withdrawal were reported over the 3-year follow-up of nusinersen therapy. Demographic and baseline clinical characteristics of the patients are presented in Table 1. The information on SMA type, *SMN2* copy number, age of onset, onset-to-injection period, and changes in motor score from baseline for all patients enrolled in this study is provided in Supplementary Table S1. At the 3-year follow-up, the analysis included 7 patients with type 1 SMA, 12 patients with type 2 SMA, and none with type 3 SMA. Nearly half of all enrolled patients across SMA types (42.8, 59.2, and 46.2%, respectively) reached the 2-year follow-up for analysis.

**Table 1:** Clinical characteristics of enrolled patients.

	Total	SMA types		
		I	II	III
Number of patients (*n*, %)	137	21 (21/137, 15.3%)	103 (103/137, 75.2%)	13 (13/137, 9.5%)
Mean treatment starting age in years (SD)	14.3 (11.2)	2.3 (4.6)	15.4 (10.0)	24.4 (12.1)
Mean symptom onset to initial injection years (SD)	13.3 (10.9)	2.1 (4.5)	14.3 (10.0)	23.0 (12.05)
Partial respiratory support (*n*, %)*	32 (32/137, 23.3%)	3 (3/21, 14.3%)	23 (23/103, 22.3%)	6 (6/13, 46.2%)
*SMN2* copy number assessment performed (n, %)	104 (104/137, 75.9%)	18 (18/21, 85.7%)	79 (79/103, 76.7%)	7 (7/13, 53.8%)
1–2 copies (*n*, %)	17 (17/104, 16.3%)	14 (14/18, 77.8%)	3 (3/79, 3.8%)	0 (0/7, 0%)
3–4 copies (*n*, %)	86 (86/104, 82.7%)	4 (4/18, 22.2%)	76 (76/79. 96.2%)	7 (7/7, 100%)
Serious adverse events	None reported			

*Needs respiratory support during daytime (8–16 h/day).

### 3.2 Motor function changes in patients with type 1 SMA

**Figure 2:**
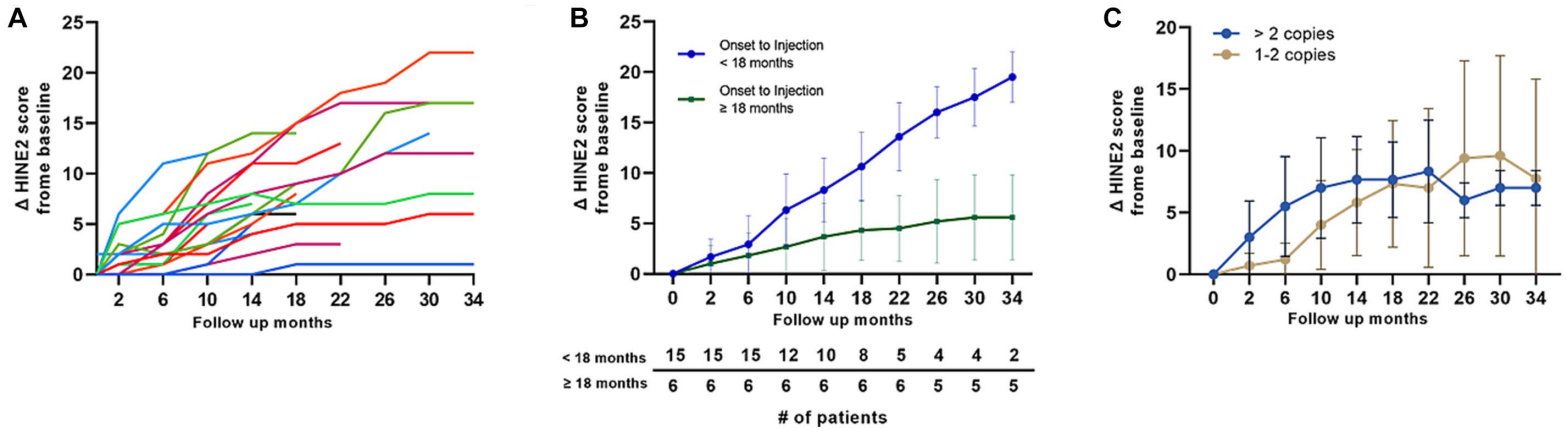
Motor score changes in patients with type 1 SMA (HINE-2). **(A)** Changes in the HINE-2 motor score in all the patients with type 1 SMA. Color codes in the graph refer to individual patients enrolled in the study. **(B)** Changes in the HINE-2 motor score from baseline in patients treated with nusinersen within 18 months of symptom onset and in patients treated 18 months after symptom onset. The number of patients at each time points are shown in the table below the graph. **(C)** Motor score changes of patients with type 1 SMA based on the number of SMN2 copies. SMA, spinal muscular atrophy, HINE-2: Hammersmith Infant Neurological Exam Section 2.

This study included 21 patients with type 1 SMA, 16 of whom completed the 1-year follow-up, 9 reached the 2-year follow-up, and 7 reached the 3-year follow-up. Figure 2A illustrates the motor score changes from baseline in all 21 patients. All patients exhibited substantial increase in motor scores during the first year of treatment (mean HINE-2 score improvement of 6.6 from baseline to the first year) and showed steady improvements during years 2 and 3 (mean HINE-2 score improvement of 3.9 from first to second year and 0.8 points from second to third year follow-up). None of the patients demonstrated a motor score decline from baseline at the 3-year follow-up.

### 3.3 Effect of treatment initiation timing on motor improvement in patients with type 1 SMA

The mean duration from symptom onset to nusinersen injection in patients with type 1 SMA was 25.4 (54.8) months. Patients who received nusinersen treatment within 18 months of symptom onset (n = 15) demonstrated a mean improvement of 8.3 points in the HINE-2 score at 1-year (n = 10), 16.0 points at 2-year (n = 4), and 19.5 points at 3-year (n = 2) follow-up. Conversely, patients who received nusinersen treatment 18 months after symptom onset (n = 6) exhibited a mean improvement of 4.0 points in the HINE-2 score at 1-year (n = 7), 5.2 points at 2-year (n = 5), and 5.6 points at 3-year (n = 5) follow-up. Motor score changes from baseline were not significant at the 1- year follow-up but indicated significant changes (*p* = 0.02, 0.03) at the 2-year and 30-month follow-ups (Figure 2B). Statistical significance was not noted at 3 years owing to small number of patients. *SMN2* gene copy numbers did not significantly influence motor function outcomes after nusinersen therapy at 1-, 2-, and 3-year follow-ups (*p* > 0.05, Figure 2C).

### 3.4 Motor function changes in type 2 SMA

**Figure 3:**
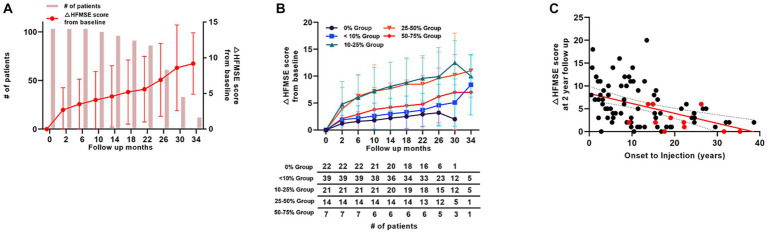
Motor score changes in patients with type 2 SMA (HFMSE). **(A)** Mean HFMSE motor score changes from baseline in patients with type 2 SMA. Bar graphs indicate the number of patients at each time point. **(B)** HFMSE motor score changes in different patient groups based on the baseline HFMSE score. The number of patients at each time points are shown in the table below the graph. **(C)** HFMSE motor score changes from baseline in patients with type 2 SMA at 2 years according to the interval from symptom onset to nusinersen injection. Patients with a HFMSE score of 0 at baseline are marked in red, and the patients with HFMSE score greater than 0 are marked in black. SMA, spinal muscular atrophy, HFMSE: Hammersmith Functional Motor Scale Expanded.

Overall, 103 patients with type 2 SMA were included in this study. Of these patients, 96 completed the 1-year, 61 reached the 2-year, and 12 reached the 3-year follow-up. The mean changes in the HFMSE scores from baseline scores are presented in (Figure 3A). For the 12 patients who reached the 3-year follow-up, the changes in mean HFMSE score from baseline scores at 1-, 2-, and 3-year were 4.7, 6.9, and 9.1, respectively. The duration from symptom onset to treatment for this group was 14.3 (10.0) years.

Across all groups of patients with type 2 SMA, only two patients (patients 23 and 88) exhibited a marked decrease in motor function after scoliosis surgery. Patient 23, with an HFMSE score of 17 at 22 months after nusinersen treatment, underwent scoliosis surgery before 26 months of follow-up, resulting in a decrease in the HFMSE score to 8 at the last follow-up (2 months after the surgery). For patient 88, the motor score of 8 at 10 months of follow-up decreased to 0 after the scoliosis operation at 14 months of follow-up. The motor scores fluctuated between 0 and 1 for 12 months after the surgery. No other patient demonstrated substantially decreased motor function over time (Supplementary Table S1).

### 3.5 Motor function changes in different baseline motor function subgroups of patients with type 2 SMA

Patients with type 2 SMA were further grouped according to their baseline HFMSE scores. Based on our subgrouping, 22 patients fell into 0% group, 39 patients into <10% group, 21 patients into 10–25% group, 14 patients into 25–50% group, 7 patients into 50–75% group, and none in the >75% group. Regarding the patients who reached the 1-year follow-up, mean HFMSE score change from baseline was 2.2 in the 0% group (n = 20), 3.0 in <10% group (n = 36), 8.1 in 10–25% group (n = 20), 7.7 in 25–50% group (n = 14), and 4.2 in 50–75% group (n = 6). The improvement incline was the lowest among all the groups in the 0% group (n = 20 at 1-year, n = 6 at 2-year, and n = 1 at 30-month follow-up). The mean increase in HFMSE score at 1-year, 2-year, and 30-month follow-ups were 4.5, 6.9, and 2.0, respectively. The apparent decline in mean HFMSE score in the 0% group was because only one patient with HFMSE score of 2 points (patient 115, Supplementary Table S1) reached the 30-month follow-up. None of the patients in the 0% group showed a decrease in HFMSE scores (Figure 3B).

**Figure 4:**
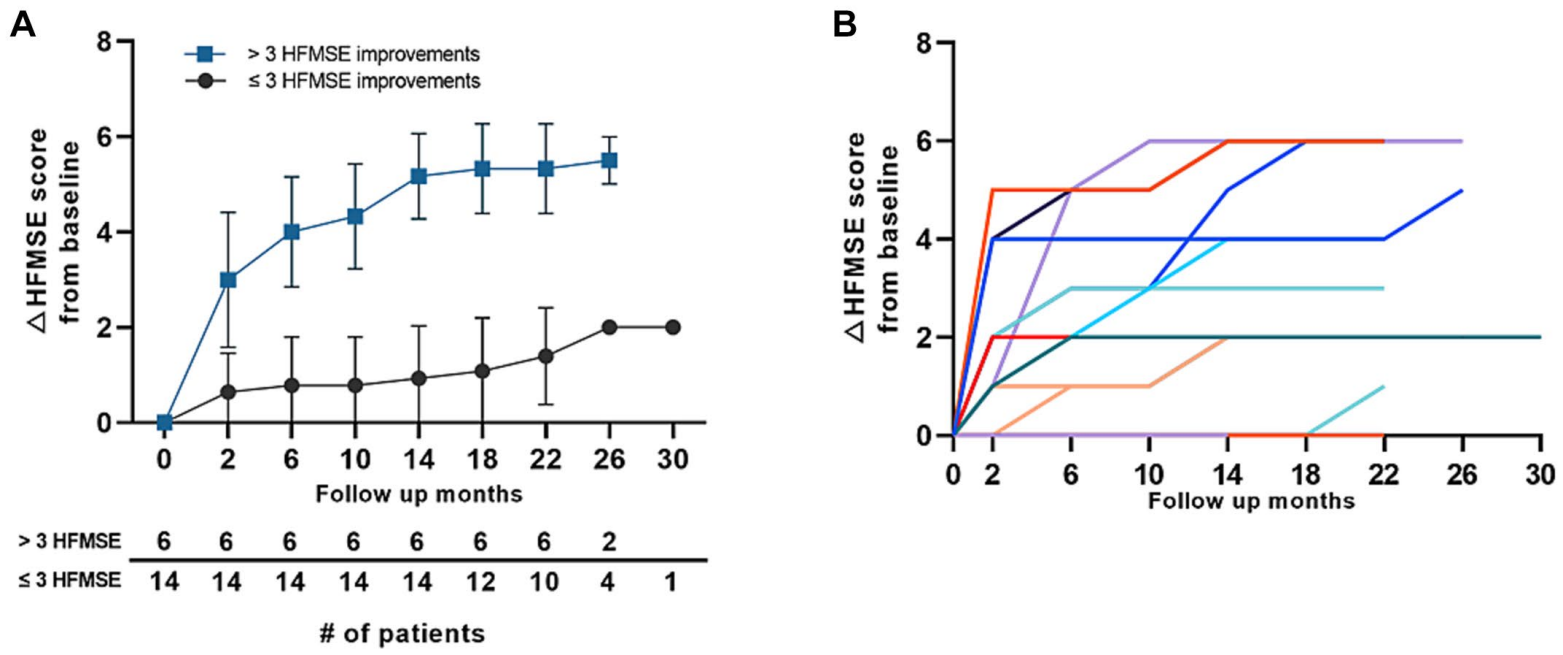
Motor score changes in the 0% group in patients with type 2 SMA (HFMSE). **(A)** Mean HFMSE motor score changes from baseline in patients with type 2 SMA with a baseline HFMSE score of 0. The patients were grouped according to ≤3 HFMSE score and > 3 HFMSE score improvements at 1 year of follow-up. Error bars depict standard deviations, and the number of patients at each time point is shown in the table below the graph. **(B)** HFMSE motor score changes in all the patients with type 2 SMA with baseline HFMSE scores of 0. Color codes in the graph refer to individual patients enrolled in the study. SMA, spinal muscular atrophy, HFMSE: Hammersmith Functional Motor Scale Expanded.

Further analysis was performed in the 0% group in which the baseline HFMSE scores were 0 before treatment with nusinersen. Of the 20 patients who reached the 1-year follow-up, 14 patients (70.0%) had ≤3 HFMSE score gain (lower 5% score), whereas six patients (30.0%) had >3 HFMSE score gain. Most patients who started with a 0 HFMSE score at baseline steadily gained motor scores throughout the follow-up period (Figure 4A). Eight patients (40%) did not gain motor scores at 1 year of follow-up, whereas 1 of the 4 patients who reached 22 months of follow-up had a point gain at 22 months (patient 97, Supplementary Table S1). Individual motor function score changes over time in patients with baseline HFMSE scores of 0 are shown in (Figure 4B). The average interval between symptom onset and the first nusinersen injection in the 0% (type 2) group was 22.9 (16.1–29.8) years. There were no significant differences in symptom to treatment interval among 0% group patients who gained greater than 3 HMFSE scores and patients who gained less than or equal to 3 HFMSE scores. Overall motor outcomes of 0% HFMSE group are provided in Supplementary Table S2.

A further comparative analysis was conducted between individuals categorized as “high” (baseline HFMSE score ≥ 35) and “low” (baseline HFMSE score < 35) in terms of baseline motor function groups. Within these groups, there were 6 patients in the high category (5.8%) and 97 patients in the low category (94.2%). The mean change in HFMSE score from baseline at the 2-year follow-up was 6.0 (SD 5.2, n = −5) for the high group and 5.6 (SD 4.5, n = 81) for the low group. No statistical differences were observed between the two groups at any time points. A summary of the high and low groups, stratified based on the baseline HFMSE scores, is provided in Supplementary Table S3.

### 3.6 Treatment initiation period in patients with type 2 SMA

The mean age of treatment initiation was 15.4 (10.0) years, and the mean period from symptom onset to nusinersen injection was 14.3 (10.0) years in patients with type 2 SMA. Among the 103 patients, only 7 were treated within 18 months of symptom onset, and 96 patients were treated after 18 months of symptom onset. Of the 61 patients who reached the 2-year follow-up, 5 patients who were treated within 18 months of symptom onset demonstrated significantly better motor score improvements (mean HFMSE score improvement = 8.4; *p* = 0.001) than the 56 patients who were treated after 18 months of symptom onset (mean HFMSE score improvement = 6.8). Figure 3C depicts an overview of the motor function improvements in all patients with type 2 SMA who reached the 2-year follow-up based on their treatment timing, demonstrating changes in the HFMSE score from the 2-year follow-up of the onset-to-injection period. A simple linear regression analysis recorded an *R^2^* value of 0.19, indicating a general tendency for worsening HFMSE scores if the onset-to-injection period was delayed. Patients with a baseline HFMSE score of 0 tended to have worse HFMSE score improvements at the 2-year follow-up (marked in red in Figure 3C).

### 3.7 Motor function changes in patients with type 3 SMA

**Figure 5:**
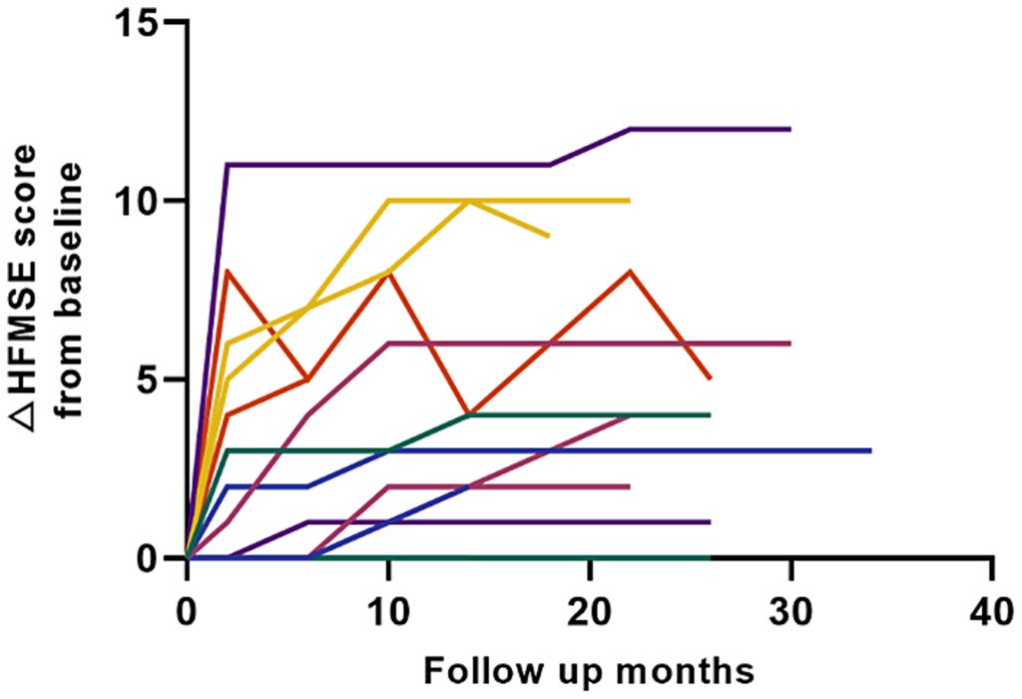
Motor score changes in all patients with type 3 SMA (HFMSE). Color codes in the graph refer to individual patients enrolled in the study. HFMSE: Hammersmith Functional Motor Scale.

Thirteen patients with type 3 SMA were enrolled in the study, six of whom reached the 2-year follow-up. The mean period between symptom onset and nusinersen treatment was 23.1 (12.0) years. Only one patient with a baseline HFMSE score of 48 demonstrated a decrease in motor function during nusinersen therapy owing to infection. However, the patient recovered well and exhibited gradual improvement in motor function (patient 135, Supplementary Table S1). All the patients with type 3 SMA demonstrated gradual improvement in motor function (Figure 5). As only six patients reached the 2-year follow-up and one patient reached the 3-year follow-up, no statistical evaluation was performed to analyze the possible risk factors for poor motor function outcomes.

### 3.8 Patient/parent-reported impressions of improvement and deterioration

The second dataset for assessment of patient/parent-reported impressions of improvement and deterioration included 115 patients (25 patients with type 1, 82 with type 2, and 8 with type 3 SMA). Analysis was performed on 108 patients (excluding with type 1 and 2 with type 2 SMA from the original dataset) who reached 1 year of follow-up. When asked the question ‘any changes in general function’ at their last follow-up, 95.0% of patients with type 1 (*n* = 19), 88.8% of patients with type 2 (n = 71), and all patients/parents of patients with type 3 SMA reported improvement in general function. One patient with type 1 and nine patients with type 2 SMA or their parents reported a stable status compared with the baseline status. Four patients with type 2 SMA who reported a stable status had a baseline HFMSE score of 0.

When asked about the specific function that improved, 50.0% of patients with type 1 (n = 10), 11.3% of patients with type 2 (n = 9), and one patient with type 3 SMA reported improvement in respiratory function. This improvement manifested as a shortened duration of nasal continuous positive air pressure support, increased comfort in respiration, enhanced volume of voice, and a newfound ability for active coughing. Eighteen patients with type 1 and 77 patients with type 2 SMA reported improved fine motor function, including increased grasp and finger movements, spooning, and ability to wear earphones. Moreover, four patients with type 1 and four patients with type 2 demonstrated improvement in speech and swallowing. A summary of these results is provided in Table 2. A summary of 3-year motor function scores for all enrolled patients is provided in Supplementary Table S4.

**Table 2:** Patient/parent-reported impressions of improvement and deterioration at 1-year follow-up.

Total patients = 108	Type 1 (*n* = 20)	Type 2 (*n* = 80)	Type 3 (*n* = 8)
Did you observe any change in general function (stable, improved, or deteriorated)?	Improved (19, 95.0%) Stable (1, 5.0%)	Improved (71, 88.8%) Stable (9, 11.3%)	Improved (8, 100%)
Which function improved as reported by patients/parents (respiratory, fine motor function, swallowing, and speech)? – Multiple answers allowed	Respiratory function (*n* = 10, 50.0%) Fine motor (*n* = 18, 90.0%) Speech/Swallow (*n* = 4, 20.0%)	Respiratory function (*n* = 9, 11.3%) Fine motor (*n* = 77, 96.3%) Speech/Swallow (*n* = 4, 5.0%)	Respiratory function (*n* = 1, 12.5%) Fine motor (*n* = 8, 100%) Speech/Swallow (*n* = 0, 0%)
Did you observe any deterioration as reported by the patients/parents (respiratory, fine motor function, swallowing, and speech)?	None reported deterioration	None reported deterioration	None reported deterioration

## 4 Discussion

This is the first long-term population-based study in South Korea and Asia-Pacific region, encompassing comprehensive data from all patients with SMA treated with nusinersen over a period of 3 years. This report demonstrated the safety and efficacy of nusinersen, along with the impressions of patients and parents regarding changes in condition with the treatment. This study incorporated all motor function data from the HIRA database, collected for the approval of nusinersen reimbursement. This comprehensive approach enabled us to present a holistic view of nusinersen efficacy in real-world scenarios.

Based on our long-term follow-up of motor function, the rate of improvement decreased over the course of treatment. In this study, the patients with type 1 SMA showed an improvement of 6.6 points in HINE-2 scores during the first year, which reduced to a 3.9-point increase during the second year. This finding aligns with previous short-term follow-up studies, which reported a mean HINE-2 score increase of 2.2–3.2 from baseline to the first year and 0.9–1.9 from the first year to the second year (12, 16–18). The plateauing of motor function improvement between years 2 and 3 (mean 0.8 HINE-2 score improvement) in this study was consistent with findings from a recently published 3-year follow-up Dutch study, which reported a mean increase of 0.6 in HINE-2 scores between years 2 and 3 (11). In patients with type 2 SMA, steady improvements in the HFMSE score were observed at 1 and 2 years of follow-up, with a mean increase of 4.7 from baseline to the first year and 2.2 in the second year of treatment. These results were consistent with those observed in previous studies, reporting mean HFMSE score changes of 2.8–4.3 at year 1 from baseline and 0.8–1.5 from the first to the second year of follow-up (3, 11, 19). From the second to the third of year follow-up, a slow but steady increase in HFMSE score (mean 0.4) was observed in 12 patients who reached 3 years of follow-up. Such improvement was similar yet distinct from the 3-year follow-up Dutch study demonstrating a plateau or a slight decrease in motor functions from 24 to 30 months of follow-up (mean 0.2 HFMSE point decrease between 24 to 30 months of follow-up) (11). Our results suggest the need for further long-term studies to elucidate the enduring treatment effects of nusinersen in patients with type 2 SMA.

Earlier initiation of nusinersen treatment in patients with type 1 SMA resulted in significantly better motor function, particularly in the long-term follow-up. The mean changes in motor scores at 6 months in each group treated within and after 18 months were 2.8 and 2.3, respectively. At the 3-year follow-up, the differences were 19.5 and 5.6 for those treated within and after 18 months, respectively, indicating clinical significance. This pattern was also observed in patients with type 2 SMA at the 2-year follow-up, which is consistent with the results of CHERISH and ENDEAR studies (1, 6). These results, along with those of previous studies, call for an increased awareness of the disease for early diagnosis and neonatal screening (20–22).

Our subgroup analysis based on the pre-treatment HFMSE score provides new insights into the efficacy of nusinersen in patients with type 2 SMA. Motor score improvement was the lowest in the 0% group, with a baseline HFMSE score of 0. At the 1-year follow-up, 12 patients (60%) in the 0% group demonstrated a mean HFMSE score increase of 3.7. The other eight patients (40%) did not have any motor score gains. Four of these patients reported a subjectively stable state without deterioration at 1 year of follow-up, while the other four reported subjective improvements in general functions. Since one of these patients gained 1 HFMSE score at 22 months, a more extended follow-up period is necessary to fully understand the long-term effects of nusinersen in patients with type 2 SMA who have the lowest baseline motor function. Moreover, considering the natural deteriorating course of type 2 SMA, this result of stable to improving motor function indicates that nusinersen has positive effects even in the lowest motor function groups of patients with type 2 SMA and is even more substantial in patients/parents’ subjective impressions. A recent study investigating the minimal clinically important difference (MCID) in patients with SMAs reported that 15 treatment naive type 2 adult patient achieved a median of 2 HFMSE scores and had relatively low MCID values (0.5–1.2) (23). However, our results indicate that 60% (12 of 20) of the patients in the 0% group gained at least 2 points at the 1-year follow-up, surpassing the MCID suggested in the aforementioned study. However, careful clinical interpretations of the HFMSE scores in 0% baseline group is needed, given the limited effectiveness of HFMSE scores in evaluating weaker SMA patients owing to its floor effects (24, 25).

Statistical significance in long-term motor function outcomes was not observed in the high (baseline HFMSE score ≥ 35) and low (baseline HFMSE score < 35) groups, contrary to findings reported in a prior study (8). This lack of significance may be attributed to the limited number of participants in the high group (n = 6, 5.8%), as compared to the larger sample size in the low group (n = 97, 94.2%), potentially introducing statistical bias. Additionally, the disparity in age distribution at the time of treatment between our cohort (mean 14.3 years, SD 11.2) and the cohort studied by Hagenacker et al. (mean 36 years, SD 12) could account for the observed differences in treatment effects. Nevertheless, our dataset yields valuable insights, emphasizing the necessity for stratification based on baseline HFMSE scores when assessing the efficacy of nusinersen on long-term motor function outcomes. A careful approach is needed to interpret the long-term motor score changes in patients with type 3 SMA in this study since only three patients reached 30 months of follow-up and one patient reached 3 years of follow-up. Congruent with other shorter follow-up studies reporting motor score changes of 1.8–4.2 HFMSE points from baseline at different follow-up periods (19, 26, 27), patients with type 3 SMA in our study also had a positive response. A follow-up study is required to determine the long-term efficacy of nusinersen in patients with type 3 SMA.

The number of patients with type 1 SMA in this study was relatively smaller (16.0%) than that of other population-based studies, including 30–50% of patients with type 1 SMA among the three types (11, 28, 29). The genetic diagnosis of SMA has significantly improved since the introduction of the multiplex ligation-dependent probe amplification method in 2006 (30). Since then, many patients with SMA received supportive care until the early access program for nusinersen became available. Considering that the National Health Insurance of South Korea covers all medical costs for patients with SMA, it is less likely that patients with type 1 SMA are underdiagnosed. The main reason for the low proportion of patients with type 1 SMA in our study was that most patients had received full respiratory support before nusinersen was available. The reimbursement criteria of the HIRA excluded patients who were under ventilator support for >16 h per day.

The patient/parent-reported impressions of improvement showed very different results from the actual motor function improvement. In this study, 90.7% of the patients and parents reported symptomatic improvement from baseline, and none reported deterioration. Improvements were more substantial in motor function than in respiratory and bulbar functions, which is consistent with recently published reports depicting fewer effects of nusinersen on improving respiratory and bulbar functions in long-term follow-ups (31–33). Such differences in patient perception and motor score changes suggest the adoption of alternative motor scales that include more detailed information on function, such as the Revised Upper Limb Module (RULM) and the Children’s Hospital of Philadelphia Infant Test of Neuromuscular Disorders (CHOP INTEND). Careful stratification of patients’ quality of life is needed to fully evaluate the status of patients over the treatment course during nusinersen treatment (34). Additionally, evaluation systems for swallowing, speech, respiration, and fine motor functions are required to determine whether there are real changes in these functions. Lessons can be learned from rating scales for amyotrophic lateral sclerosis such as the Amyotrophic Lateral Sclerosis Functional Rating Scale-Revised which encompasses symptoms such as dyspnea, orthopnea, and respiratory insufficiency. Adding a subscale for these categories could add values in evaluating patients with very low motor scores in various scales (35, 36).

No serious adverse events requiring withdrawal of nusinersen treatment were reported in this study. Anesthesia and/or sedation for nusinersen injections were well tolerated, with no reported side effects. Fluoro-guided transforaminal injections were administered to patients with severe scoliosis or previous spinal surgery without complications.

This study had some limitations. First, detailed clinical information such as scoliosis state, specific motor milestones, neurodevelopmental status, feeding state, and respiratory and bulbar functions, was lacking. Such limitations were due to anonymization of the enrolled patients. In a recent study, respiratory and bulbar functions did not improve markedly during nusinersen treatment (31). The lack of detailed clinical data limited our study from providing a clear clinical picture of our large cohort. Second, only the HFMSE and HINE-2 scores were used to evaluate motor function. Scales such as the RULM and/or CHOP INTEND could have assisted our study in analyzing the improvement in motor, respiratory, and bulbar functions more accurately. Finally, the inclusion of only 19 patients reaching the 3-year follow-up, constituting 12.4% of the enrolled participants, imposes limitations on our study’s capacity to present comprehensive long-term motor function results for the entire 3-year follow-up period.

Nevertheless, this study has several strengths. First, to the best of our knowledge, this is the first 3-year follow-up population-based study of all SMA types from the Asia-Pacific region, with the largest population-based patient group to date. Second, detailed long-term motor function trends in all types of SMA and different baseline motor function groups in patients with type 2 will provide strong real-world evidence that could be used in various clinical situations. Increasing roles of real-world data in the regulatory processes of rare diseases highlight the importance of population-based studies (37, 38). As there are more debates on the cost-effectiveness of nusinersen (39–41), these real-world data indicating different pattern of improvement according to the treatment timing and baseline motor function in long-term outcomes of patients with type 1 and 2 SMA will have high clinical importance. Congruent results in our patient/parent-reported assessments of minimal bulbar/respiratory function improvements in various studies call for detailed evaluation strategies during the follow-up of patients with SMA. Lastly, this study provides large, long-term follow-up results with significant evidence confirming that earlier treatment with nusinersen after symptom onset is most beneficial. This evidence strongly supports the implementation of SMA in newborn screening.

Nusinersen treatment in patients with types 1–3 SMA is safe and effective in long-term follow-up. Early initiation of treatment after symptom onset had the most significant effect. Further detailed studies are needed to evaluate the long-term effects of nusinersen on respiratory and bulbar functions as well as on the quality of life of patients with SMA.

## Data availability statement

The original contributions presented in the study are included in the article/Supplementary material, further inquiries can be directed to the corresponding author.

## Ethics statement

The studies involving humans were approved by Institutional Review Board (IRB) of Seoul National University Hospital, Institutional Review Board (IRB) of Korean Health Insurance Review and Assessment Service, and Institutional Review Board (IRB) of Samsung Medical Center. The studies were conducted in accordance with the local legislation and institutional requirements. Written informed consent for participation was not required from the participants or the participants’ legal guardians/next of kin in accordance with the national legislation and institutional requirements.

## Author contributions

JC: Conceptualization, Data curation, Formal analysis, Investigation, Methodology, Project administration, Writing – original draft. JiL: Conceptualization, Data curation, Formal analysis, Investigation, Methodology, Project administration, Writing – original draft, Writing – review & editing. JK: Conceptualization, Data curation, Formal analysis, Investigation, Methodology, Project administration, Writing – original draft, Writing – review & editing. HL: Data curation, Supervision, Writing – review & editing. M-JK: Data curation, Supervision, Writing – review & editing. YL: Data curation, Supervision, Writing – review & editing. M-SY: Data curation, Supervision, Writing – review & editing. J-HB: Data curation, Supervision, Writing – review & editing. CL: Data curation, Supervision, Writing – review & editing. Y-ML: Supervision, Writing – review & editing, Data curation. JeL: Conceptualization, Funding acquisition, Methodology, Project administration, Resources, Supervision, Validation, Writing – original draft, Writing – review & editing. JC: Conceptualization, Funding acquisition, Methodology, Project administration, Resources, Supervision, Validation, Writing – original draft, Writing – review & editing.
